# The Apparent Involvement of ANMEs in Mineral Dependent Methane Oxidation, as an Analog for Possible Martian Methanotrophy

**DOI:** 10.3390/life1010019

**Published:** 2011-11-18

**Authors:** Christopher H. House, Emily J. Beal, Victoria J. Orphan

**Affiliations:** 1 Department of Geosciences and Penn State Astrobiology Research Center, The Pennsylvania State University, 220 Deike Building, University Park, PA 16802, USA; E-Mail: ejbeal@gmail.com; 2 Division of Geological and Planetary Sciences, California Institute of Technology, Pasadena, CA 91125, USA; E-Mail: vorphan@gps.caltech.edu

**Keywords:** Archaea, methane, methanotrophy, Mars, subsurface biosphere

## Abstract

On Earth, marine anaerobic methane oxidation (AOM) can be driven by the microbial reduction of sulfate, iron, and manganese. Here, we have further characterized marine sediment incubations to determine if the mineral dependent methane oxidation involves similar microorganisms to those found for sulfate-dependent methane oxidation. Through FISH and FISH-SIMS analyses using ^13^C and ^15^N labeled substrates, we find that the most active cells during manganese dependent AOM are primarily mixed and mixed-cluster aggregates of archaea and bacteria. Overall, our control experiment using sulfate showed two active bacterial clusters, two active shell aggregates, one active mixed aggregate, and an active archaeal sarcina, the last of which appeared to take up methane in the absence of a closely-associated bacterial partner. A single example of a shell aggregate appeared to be active in the manganese incubation, along with three mixed aggregates and an archaeal sarcina. These results suggest that the microorganisms (e.g., ANME-2) found active in the manganese-dependent incubations are likely capable of sulfate-dependent AOM. Similar metabolic flexibility for Martian methanotrophs would mean that the same microbial groups could inhabit a diverse set of Martian mineralogical crustal environments. The recently discovered seasonal Martian plumes of methane outgassing could be coupled to the reduction of abundant surface sulfates and extensive metal oxides, providing a feasible metabolism for present and past Mars. In an optimistic scenario Martian methanotrophy consumes much of the periodic methane released supporting on the order of 10,000 microbial cells per cm^2^ of Martian surface. Alternatively, most of the methane released each year could be oxidized through an abiotic process requiring biological methane oxidation to be more limited. If under this scenario, 1% of this methane flux were oxidized by biology in surface soils or in subsurface aquifers (prior to release), a total of about 10^20^ microbial cells could be supported through methanotrophy with the cells concentrated in regions of methane release.

## 1. Introduction

The past decade has seen remarkable progress in the understanding of the microbiology of methane consumption in marine sediments and seeps. We have learned that marine sulfate-dependent anaerobic oxidation of methane (AOM) is performed by at least three groups of archaea: ANME-1, ANME-2, and ANME-3 [1,2,3,4,5,6,7,8]. The ANME, in general, are related to methanogenic archaea, and there is also growing evidence that some ANME can reverse their metabolism, producing methane at times [9,10,11,12]. Both ANME-2 and ANME-3 are usually found in organized aggregates with sulfate reducing bacteria (mainly associated with *Desulfosarcina/Desulfococcus* and *Desulfobulbaceae*) indicating that methane oxidation by ANME archaea is often coupled directly or loosely to a syntrophic sulfate-reducing bacterial partner [1,2,13]. In freshwater environments, nitrite and nitrate have also been shown to be possible electron acceptors for methane oxidation [14,15]. A novel methylotrophic bacterium (Candidatus ‘Methylomirabilis oxyfera’) has recently been linked to this process [16].

In general, it appears that methane oxidation can be coupled to a variety of favorable electron acceptors including metal oxides [17,18]. The presence of ^13^C-depleted carbonates suggest that manganese-dependent methane oxidation leads to the formation of rhodocrosite (MnCO_3_) deposits, throughout the California Coast Range [19]. Both manganese and iron enrichment in methane associated carbonates have also been observed in the Black Sea [20] and Eel River Basin [21]. In laboratory incubations with methane seep sediment, Beal *et al.* [17] found that manganese, in the form of birnessite, and iron, in the form of ferrihydrite, can be used in marine AOM in the absence of sulfate [17], converting ^13^CH_4_ into ^13^CO_2_. Here, we provide new data from methane seeps on Earth that indicate that the microbial cells involved with manganese-associated AOM in marine sediments are similar to those involved with sulfate-dependent AOM. The result suggests that the same cells can grow through methane oxidation in the presence of both sulfate and metal oxides.

With the recent discovery of significant seasonal methane outgassing on Mars, methane oxidation coupled to the reduction of minerals (sulfates and metal oxides) represents a plausible set of metabolisms for supporting a Martian microbiota, present and past. Here, based on the previous studies of these and similar microbial consortia, we estimate the potential size of a global Martian methanotrophic community using the growth parameters developed from this Earth-based system with the understood assumption that microbes in a Martian methanotrophic ecosystem are analogous to that of the methane-oxidizing ANMEs.

## 2. Methods

All analyzed samples came from the ^13^C-labled methane incubation studies described by Beal *et al.* [22]. The methane seep sediment samples used in the incubations came from the Eel River Basin, CA (ERB) collected in August 2005 using the R/V *Western Flyer*. As previously reported, incubations contained methane seep sediment, artificial seawater [23], ^13^C-enriched methane, carbon dioxide, and an added electron acceptor (sulfate or birnessite). At the end of the 10-month experiments, one bottle from the manganese experiment series and one bottle from the sulfate experiment series were amended with ^15^NH_4_Cl at a final concentration of 2 mM.

The experiments were incubated at 10 °C for an additional three weeks with the ^15^NH_4_Cl and then sampled (1 mL sediment/sea water slurry) for FISH-SIMS analysis to determine which target aggregates incorporated ^15^N during *de novo* protein synthesis (and thus are active in our incubations) [24]. The sediment sampled was washed three times using 1.5 × phosphate-buffer saline (PBS). Samples were then fixed by adding 750 μL 4% (wt/vol) paraformaldehyde and 250 μL 1.5 × PBS and incubating for 3 hours at 4 °C. After fixation, samples were washed using 1.5 × PBS and then stored at −20 °C in a 1:1 ethanol/PBS solution. Aggregates were separated from sediment on a Percoll density gradient at 4 °C as outlined by Orphan *et al.* [2]. The entire Percoll gradient (with the exception of the sediment pellet) was then filtered onto a 3 μm polycarbonate filter, 25 mm in diameter (Millipore, Billerica, MA, USA). Cells were transferred to a 1 inch glass round [1], followed by a ethanol dehydration series (50%, 70%, and 100% ethanol, diluted with 1 × PBS).

The protocol for FISH [25] was conducted on samples using oligonucleotide probes ARCH915 and EUB338_MIX (labeled with either CY3 or FITC, Sigma Oligo). Prior to viewing and mapping, hybridized slides were marked using a diamond pen to facilitate locating SIMS targets as described in Orphan *et al.* [1]. The slides were then counterstained with DAPI combined with a water-soluble glycerol and PBS mounting medium (at pH = 8.0). Aggregates were identified using a 60× oil immersion objective (Olympus PlanApo). Both epiflourescent and transmitted light images, as well as epiflourescent “z-stacks” were collected using a DeltaVision RT deconvolution microscope system and the image analysis software SoftWoRx version 3.5.1 [26]. The locations of observed hybridized aggregates were recorded for subsequent identification and analysis by SIMS as described in Dekas and Orphan, 2011. After identifying aggregates, the DAPI/mounting medium was gently removed using distilled water. Mapped aggregates were revisited and transmitted light images were collected at lower magnification (40× or 10×; Olympus objective UPlanApo). Low magnification (10×) paneled images were also collected for areas of the slide containing target aggregates and reference marks [27]. These images were then used for locating target aggregates with the optical camera system on the CAMECA 1,270 ims ion microprobe.

We used the UCLA CAMECA 1270 IMS ion microprobe in a multi-collection mode to measure the carbon and nitrogen isotopic composition of positively identified archaeal/ bacterial aggregates as described in Orphan *et al.* [24]. The instrument was configured to use electron multipliers to simultaneously collect ^12^C^13^C^−^ (on-axis) and ^12^C_2_^−^ (off-axis). The magnet was cycled to use the on-axis detectors to collect counts for ^12^C^14^N^−^ and ^12^C^15^N^−^. The primary Cs^+^ beam (typically 0.1 to 0.3 nA) had a spot size of approximately 15 μm. A correction for instrumental fractionation was determined by measuring *Escherichia coli* cells, with known carbon and nitrogen isotopic compositions, on the SIMS [2,24]. Typical analytical precision on carbon and nitrogen isotopic measurements was less than 2‰ and 10‰ respectively. In a parallel non-active control, we observed cell clusters with δ^15^N up to about 150‰ after incubation with ^15^NH_4_Cl, presumably due to abiotic absorbance of the ^15^N labeled substrate. Therefore, for this study, we used a cutoff of δ^15^N ≈ 150‰ as the threshold for assigning a target aggregate as metabolically active.

## 3. Results

### 3.1. Fluorescence *In Situ* Hybridization

**Figure 1:**
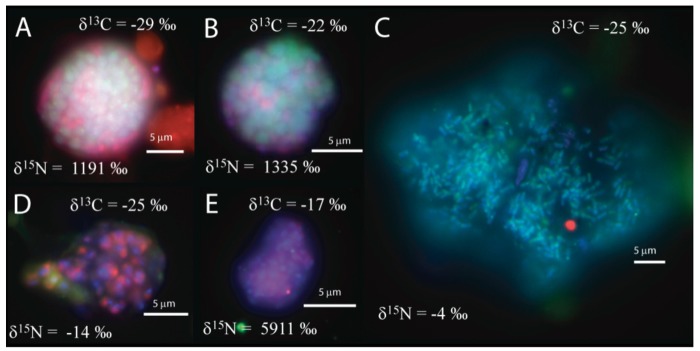
FISH images of the sulfate incubation showing (**A**) a shell aggregate, (**B**) a mixed aggregate, (**C**) a cluster of archaea rods (morphologically similar to ANME-1), (**D**) and (**E**) bacterial clusters. Cy3 (red) is bacteria, FITC (green) is archaea, and all images also contain DAPI (blue). High δ^15^N values indicate active cells with *de novo* protein synthesis during incubation with sulfate and methane.

We observed that the sulfate incubation, run in parallel with the manganese incubation, contained shell aggregates, with an archaeal core surrounded by bacteria (3 of 14 mapped aggregates), such as Figure 1a, as well as one mixed aggregate (Figure 1b). In addition, one large archaeal aggregate (20 μm by 35 μm) morphologically resembling the rod-shaped ANME-1 [1,28], Figure 1c), 3 clusters of archaea with a sarcina morphology, and small clusters (5–10 μm) of bacteria (Figure 1d–e) were recovered. The variety of observed aggregates in our sulfate incubation, are in good agreement with reports from other methane seep environments [1,2,3,4,5,7,24].

**Figure 2:**
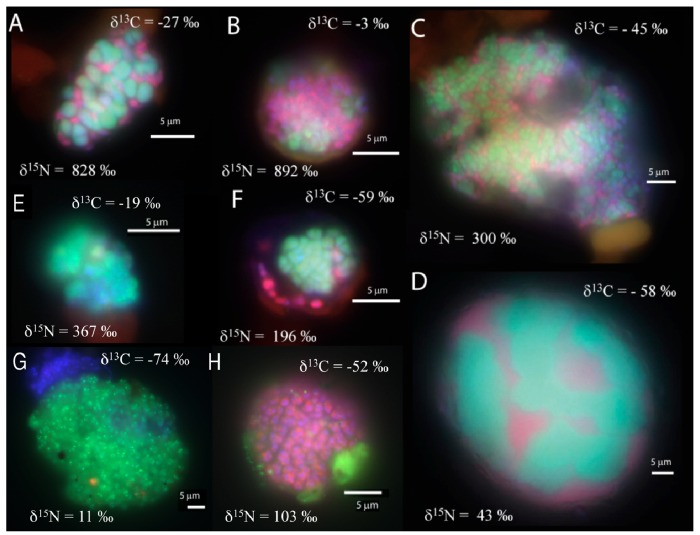
FISH images of cells from the manganese incubation showing (**A–D**) mixed and mixed-cluster aggregates, (**E**) an archaeal aggregate of sarcina morphology, (**F**) a shell aggregate, (**G**) archaea cocci, and (**H**) a bacterial cluster. Cy3 (red) is bacteria, FITC (green) is archaea, and all images also contain DAPI (blue). High δ^15^N values indicate active cells with *de novo* protein synthesis during incubation with manganese and methane.

In the manganese incubation, mixed (and mixed cluster) aggregates of bacteria and archaea appeared to be the common morphology (8 of 33 screened aggregates), with diameters ranging from 10 to 40 μm. (Figure 2a–d). A few shell aggregates (3 of 33 screened aggregates), similar to those in the sulfate incubation, were also present (Figure 2f). Additionally, archaeal clusters of sarcina morphology (2 of 33 screened aggregates; Figure 2e) were observed. Aggregates of small archaeal cocci (such as Figure 2g) were also detected, (3 of 33 screened aggregates, 10-30 μm aggregate diameter). Many bacterial clusters (such as Figure 2h), ranging in size from a few μm up to 50 μm diameter, were also detected. In general, cell aggregates in the manganese incubation tended to be larger than those detected in the associated sulfate incubation, with 15 of the 33 screened aggregates having a diameter greater than 15 μm.

### 3.2. ^13^C/^15^N analysis of Archaeal-Bacterial Aggregates by FISH-SIMS

**Figure 3:**
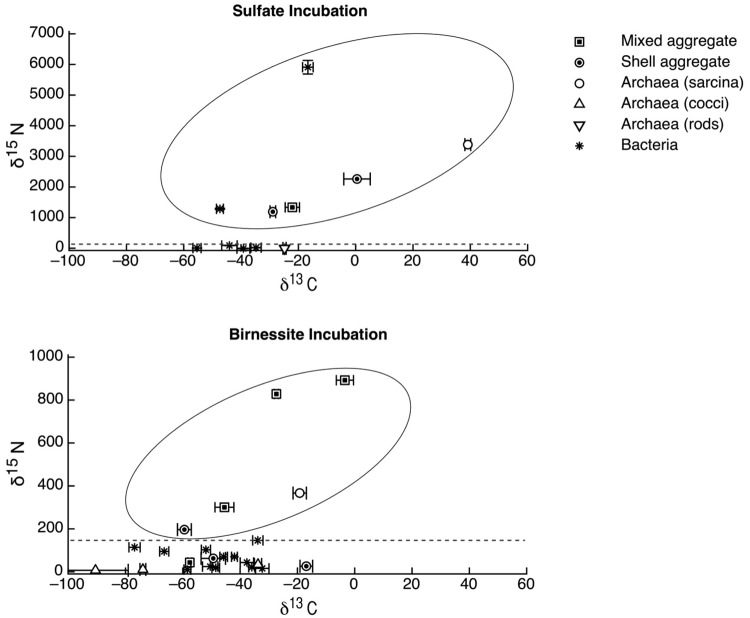
Carbon and nitrogen isotopic compositions of targeted aggregates. Aggregates classification scheme based on description in text as well as indicated in Figure 2 and Figure 3. Dashed line shows the approximate maximum ^15^N values observed in the non-active control incubation (∼150‰). The top panel shows aggregates from the sulfate control. The bottom panel shows aggregates from the manganese incubation. Shell aggregates are active in the sulfate incubation, but not active in the manganese as indicated by incorporation of ^15^N. Mixed/mixed cluster and archaea sarcina are active in the manganese incubation (as shown by incorporation of ^15^N). Aggregates in the manganese (birnessite) incubation (bottom panel) are less active than those in the sulfate incubation (top panel) as expected due to the slower rate of manganese dependent AOM as compared to sulfate dependent AOM. Typical carbon isotopic composition of shell and mixed and mixed-cluster aggregates in the ERB range from −100 to −60‰ (although some have been found as enriched as −20‰). Archaea sarcina have typical ^13^C values ranging from −80 to −20‰. Bacteria are typically heavier, with ^13^C values typically between −45‰ and −20‰ [1,2,9]. Because we do not know the ^13^C value of the target aggregates before incubation with ^13^CH_4_, we cannot conclusively state which aggregates show minor incorporation of ^13^C. However, our data indicate that the more active aggregates (shown in an oval) are also slightly enriched in ^13^C as compared to other active aggregates in our incubation, suggesting that they have incorporated minor amounts of ^13^C from methane.

The carbon and nitrogen isotopic composition was measured by SIMS for the aggregates from the manganese incubation and its sulfate control. A total of 10 FISH identified aggregates from the sulfate incubation and 21 aggregates from the manganese incubation were measured for carbon and nitrogen isotopes. SIMS results for the sulfate incubation indicated that aggregates with both shell and mixed morphologies, in most cases, have the largest amount of ^15^N incorporation after the 3-week incubation period with nitrogen-15 labeled NH_4_ (Figure 3a). In addition to the archaeal/ bacterial consortia, one mono-specific bacterial cluster was also observed to be highly enriched in ^15^N (Figure 3a). However, most of the targeted mono-specific bacterial clusters (4 of 5) showed virtually no or minor incorporation of ^15^N relative to cells in the control incubation. In contrast, only one archaeal / bacterial shell aggregate in the manganese incubation showed significant ^15^N incorporation (Figure 3b). Monospecific archaeal and bacterial clusters also showed minor ^15^N enrichment, at a much lower level than similar cell types analyzed from the sulfate incubation. Mixed / mixed cluster archaeal/bacterial aggregates, in addition to archaeal aggregates of sarcina morphology, show more ^15^N incorporation than all of the other targeted aggregates in the manganese incubation (Figure 2a–f, Figure 3b). Surprisingly, the larger aggregates found in the manganese incubation (such as Figure 2c–d) showed less ^15^N incorporation than the smaller aggregates. The two targeted mixed/ mixed cluster aggregates greater than 20 μm in diameter had δ^15^N = 43‰ and δ^15^N = 300‰, while the δ^15^N of the smaller mixed and mixed-cluster aggregates were 830‰ and 890 ‰. While these δ^15^N values strongly indicate that these cells were active, the actual amount of incorporation of the label over the 3 week incubation period is relatively small (less than 1% of the nitrogen in the biomass is label), and considerably less than was observed for similar sulfate incubations by Orphan *et al.* [23] and Dekas *et al.* [29] for both the Mn and SO_4_ treatments. This rate of ^15^N-incorporation (measured after a 10-month incubation without ^15^N-label) implies doubling times of greater than four years for the archaeal/bacterial consortia in our incubation (compared with 3–6 months doubling times estimated for SO_4_-dependent AOM [24,30,31]). These results suggest that after this long time interval, the cells, while viable and full of intact ribosomal RNA, were in a stationary growth phase.

Because of the 10-month incubation period with ^13^CH_4_, the carbon isotopic composition of all target aggregates was also measured to assess ^13^C incorporation indicative of methanotrophy. Shell and mixed aggregates in the sulfate incubations have δ^13^C values ranging from −29 to −0.5‰ (Figure 3a). The δ^13^C of bacterial clusters, range from −47 to −16.5‰. Archaea (rods, sarcina, and cocci) range from δ^13^C = −25 to +39‰. Mixed aggregates in the manganese incubation have δ^13^C values ranging from −49.5 to −3.5‰ (Figure 3b). Shell aggregates are slightly less enriched in ^13^C, with δ^13^C values ranging from −73 to −17‰. Archaea (sarcina and cocci) have δ^13^C = −90.5 to −19‰. Bacteria range from δ^13^C = −78 to −34‰. While it is difficult to use ^13^CH_4_ incorporation to estimate growth rates because other carbon sources are likely utilized during growth [32], the δ^13^C results are also consistent with ^15^NH_4_ data suggesting extremely slow growth rates under the incubation conditions used.

## 4. Discussion

### 4.1. SIMS Isotope Tracer Study of Manganese AOM

Due to the uptake of the ^15^N-labled ammonium during transcription and protein synthesis, from the ^15^NH_4_Cl injected into the cultures in the last three weeks of the incubations, active cells in the incubation are enriched in ^15^N as compared to the inactive cells, as revealed by measuring the nitrogen isotopic composition of cell aggregates. This method has successfully been used in previous AOM incubation studies to assess activity and potential growth rates [24].

We also measured the carbon isotopic values to help determine which of the active aggregates were the ones that were also metabolizing the ^13^C-labeled methane, and thus had incorporated ^13^C into their biomass during the entire 10-month incubation period. Due to the extremely low rate of carbon assimilation by methanotrophs, estimated between 1–3% of methane oxidized [31,32,33,34,35] very little of the ^13^CH_4_ was incorporated into cells, making it difficult to distinguish between aggregates with and without ^13^C incorporation. However, it is possible to determine, in some cases, which cell aggregates show minor ^13^C incorporation when we compare the values of aggregates from our ^13^CH_4_ incubations to those occurring naturally in ERB methane seeps. Shell and mixed aggregates from the ERB seeps typically have δ^13^C values ranging from −100 to −60 ‰ [1,2], although some can be as heavy as −25‰ [9]. Archaeal aggregates of sarcina morphology have typical δ^13^C ranging from −80 to −20 ‰ [9]. However, bacteria are typically enriched in ^13^C, with δ^13^C values typically between −45‰ and −20‰ [1,2,9]. Measured cell aggregates that are more enriched in ^13^C than these natural values have likely incorporated labeled ^13^C-methane. Our SIMS analysis of the archaea found in the sulfate incubation (including shell and mixed aggregates) showed that they are active in our incubation, as indicated by the incorporation of ^15^N (Figure 2a). The carbon isotopic analyses of these archaea suggest that a subset of these microorganisms are also metabolizing methane (Figure 2a), as at least two of them are clearly enriched in ^13^C with values as high as −0.5 and +40‰ (Figure 2a). The bacteria in the sulfate incubation show little ^15^N incorporation, with the exception of one bacterial cluster (Figure 2a). However, the bacterial clusters have carbon isotopic compositions in the range of bacteria reported from the ERB, suggesting that they are not directly metabolizing methane. These results are in good agreement with other sediment incubations with methane and sulfate [24,30,31], which have typically shown the growth of ANME-2 shell clusters under AOM conditions.

The bacterial clusters from the manganese amendment, which likely include heterotrophic manganese reducers [17], also showed little incorporation of ^15^N, indicating that they were comparably less active during the incubation relative to incubations with sulfate. In addition, the bacterial clusters show similar carbon isotopic values as bacteria found in methane seep environments (Figure 3b), indicating that mono-specific bacterial aggregates were likely not involved in methane metabolism. As our SIMS analysis targeted only large bacterial clusters, we cannot rule out the possibility that individual bacteria (or small clusters) are involved in directly mediating manganese dependent AOM, but the results at this time do not support such a role for bacteria in the marine seep environment. These results suggest that bacteria alone cannot mediate manganese dependent AOM. SIMS analysis of archaeal/bacterial shell aggregates in the manganese incubation appeared to be relatively inactive (Figure 2b), as indicated by the minor incorporation of ^15^N (δ^15^N < 200 ‰) relative to the sulfate incubation. In contrast, anabolic activity by the mixed and mixed-cluster archaeal / bacterial aggregates appeared to be stimulated in the manganese incubation, showing δ^15^N values of >300 ‰ (Figure 2a–d, Figure 3b). Surprisingly, two mono-specific archaeal aggregates of sarcina morphology, resembling members of the ANME-2, show substantial incorporation of ^15^N, and appear to be metabolically active independent of a physically-associated bacterial partner. These results taken together indicate that AOM-based on the reduction of manganese can be mediated by ANME archaea either directly or through the availability of trace sulfate from metal-dependent sulfide oxidation. While different microbial aggregate morphologies were observed to be metabolically active in the different incubations, the ANME-associated archaea were clearly active in both incubations. This result suggests that the microbial cells capable of mineral-based AOM can utilize different electron acceptors (e.g., sulfate, iron oxides, and manganese oxides).

### 4.2. The Potential of Mars Methanotrophy

During the past decade, several different research groups have reported the detection of methane in the atmosphere of Mars [36,37,38,39,40]. For example, Mars Express revealed a weak methane feature at the CH_4_ Q-branch with differing intensity during differing years [36], and ground-based observations using high-dispersion infrared spectrometers have shown variable methane signals over three Martian years [39]. This later study includes the report of a large 2003 release of methane (19,000 metric tons) in the Martian Northern Summer [39]. Irrespective of whether or not the methane on Mars is biogenic, the seepage of such methane in large seasonal plumes provides the potential for methanotrophy because the Martian surface is quite oxidized and may have, in places, liquid water in the regolith [41]. The surface of Mars includes deposits of sulfates (gypsum and jarosite), phyllosilicates, iron oxides, and manganese oxides [42,43,44,45,46,47,48].

From the new work here, we can conclude that syntrophic AOM consortia demonstrate versatility in the laboratory-based incubation studies, which in turn suggests that similar groups of microorganisms could couple methane to both the reduction of sulfates and metal oxides under environmental conditions. This apparent metabolic flexibility of ANMEs provides the basis for exploring the possibility that methane could fuel methanotrophy coupled to multiple oxidized substrates on Mars. When using terrestrial microbial ecosystems as analogs for considering a Martian biosphere, it is important to be clear that there is much that we do not understand about the energetics of AOM on Earth. When mediating AOM, ANMEs are using a metabolism that is on the edge of what is considered viable for biological energy conservation [49]. So, while ANMEs certainly live in a very different setting than that of the Martian crust, they represent an interesting Earth-analog for potential Martian microorganisms. However, studies of Earth's subsurface marine sediment have shown that deeply buried zones of methanotrophy contain higher cells counts than would be expected based on typical microbial energy conservation [50]. Based on these observations, subsurface microbes might have strategies that allow them to use substantially less energy and therefore persist over long timescales on very little energy [50,51,52,53]. If this is the case, using methane seep microbiota as an analog for Martian microbiota would result in cell density estimates that are lower than could be supported with such alternative strategies of survival and cell maintenance. With this caveat in mind, here we have used data for ANME population abundance and activity in marine methane seeps to estimate the standing population of methanotrophs that could be supported given the potential methane flux on Mars.

In the most optimistic scenario, Martian methanotrophy consumes much of the periodic methane released to the Martian surface. For example, Mumma *et al.* [39] reported a large methane plume of about 19,000 metric tons during the northern midsummer 2003. In an optimistic view of life on Mars, methanotrophy would provide the necessary methane sink to explain the variable methane observed in the Martian atmosphere. Oxidation of the large plume reported for summer 2003 would consume about the amount of oxidant produced yearly through the escape of hydrogen from the Martian atmosphere, and so here we will consider such a yearly event of that magnitude as a reasonable upper limit for the long-term flux of methane to the atmosphere [54]. Doing so avoids presenting a model which is inconsistent with the present oxidized Martian surface [55]. Assuming that the long-term yearly average release is about the same as the large plume reported for summer 2003 (19,000 metric tons), the optimistic view of life of Mars is that the entire oxidation of the plume is biologically mediated in Martian soils [56]. Using marine methane seep ANME microbiota as an analog, we can look at the size of the standing population of microbes supported by a particular methane flux to determine what size of microbial population might be possible on Mars. Radiotracer studies of the zone of peak methanotrophy in a sediment core from the Gulf of Mexico (Site GC232) showed a standing population of 4.9 × 10^8^ ANME cells per cm^3^ of sediment mediating the oxidation of 3.7 × 10^−5^ moles CH_4_ per year [10]. This environmental rate of AOM suggests that about 1.3 × 10^13^ ANME cells can be supported per mole of methane oxidized annually.

This estimate is broadly consistent with laboratory rates under constant methane pressure, which show about 6.8 × 10^9^ ANME cell aggregates can be supported per mole of methane oxidized annually [31]. Assuming 1.3 × 10^13^ ANME cells can be supported per mole of methane oxidized annually and assuming about 1.2 × 10^9^ moles of methane are released annually on Mars, the yearly plumes would support a standing Martian population of 1.5 × 10^22^ methanotrophs, corresponding to about 10,000 cells per cm^2^ on Mars in a zone of methanotrophy very close to the surface (in the regolith). As pointed out in Zahnle *et al.* [55]'s discussion of the difficulties of having long-term variable methane on Mars, cyclic biological production and destruction of methane in shallow subsurface Mars would be one way to explain the observation of methane variability because this process would not fully deplete all of the oxidants on the Martian surface over time. This carbon cycle would allow for a stronger flux of methane and therefore support a higher cell population than calculated above. For this to occur, however, the methane would have to be produced from decomposing methanotrophic biomass at a very shallow depth. This scenario seems unlikely because it would require the whole-scale movement of methanotrophic biomass away from oxic conditions via the flow of ground water. Given a deep origin for the Martian methane, shallow methanotrophy could still be the sink for the methane released. In this scenario, long-term methane release needs to be limited to the hydrogen escape rate or methanotrophy would, over geologically-relevant time scales, consume oxic minerals on the surface and in the regolith [55]. In general, Zahnle *et al.* [55] argue against a biological sink for the large methane plumes because 800 ppmv carbon monoxide is seen in the Martian atmosphere. Unless this carbon monoxide is also variable, which remains a possibility [57], this would seem to suggest that a diverse microbial community is not in contact with the atmosphere. We have previously observed that AOM is inhibited by high concentrations of carbon monoxide [58]. While we do not know if this inhibition is due to carbon monoxide being a competitive substrate or if it is due to carbon monoxide inhibition of Methyl-CoM reductase, our current working hypothesis for the ANME-2 predicts that carbon monoxide would be preferentially consumed over methane when available [58].

If biological methane-oxidation is not serving as the major sink for methane released to the Martian atmosphere, it is also worth considering a subsurface biosphere based on methanotophy. Most of the methane released each year is probably oxidized through an abiotic process with a long-term release rate that is similar to the rate of hydrogen escape. In this case, some small portion of the methane traveling up through the crust would be oxidized before it reaches the atmosphere. If under this scenario, 1% of this methane flux were oxidized by biology in surface soils or in subsurface aquifers (prior to release), an estimated 10^20^ microbial cells could be supported through methanotrophy planet-wide.

## 5. Conclusion

Through FISH and FISH-SIMS analyses using ^13^C and ^15^N labeled substrates, we find that the most active cells during manganese-dependent AOM are primarily mixed and mixed-cluster aggregates of archaea and bacteria. These results suggest that the microorganisms (e.g., ANME-2 archaea) observed to be capable of maintaining metabolic activity in the manganese incubations appear to be similar to those archaeal/bacterial aggregates mediating sulfate-dependent AOM. Similar flexibility in available electron acceptors for Martian methanotrophs would mean that the same major groups of microorganisms could inhabit a diverse set of Martian mineralogical crustal environments. In an optimistic scenario, Martian methanotrophy may consume much of the periodic methane released, supporting on the order of 10,000 microbial cells per cm^2^ of the Martian surface. In a more limited scenario, where most of the methane released each year is oxidized through an abiotic process, assuming only 1% of this methane flux were oxidized by microorganisms in surface soils or in subsurface aquifers (prior to release), a total of about 10^20^ microbial cells could be supported through the anaerobic oxidation of methane planet-wide.
